# Adipose tissue mitochondrial dysfunction in human obesity is linked to a specific DNA methylation signature in adipose-derived stem cells

**DOI:** 10.1038/s41366-018-0219-6

**Published:** 2018-09-27

**Authors:** Miriam Ejarque, Victoria Ceperuelo-Mallafré, Carolina Serena, Elsa Maymo-Masip, Xevi Duran, Angels Díaz-Ramos, Monica Millan-Scheiding, Yaiza Núñez-Álvarez, Catalina Núñez-Roa, Pau Gama, Pablo M. Garcia-Roves, Miquel A. Peinado, Jeffrey M. Gimble, Antonio Zorzano, Joan Vendrell, Sonia Fernández-Veledo

**Affiliations:** 1 Hospital Universitari de Tarragona Joan XXIII—Institut d´Investigació Sanitària Pere Virgili-Universitat Rovira i Virgili, Tarragona, Spain; 2 CIBER de Diabetes y Enfermedades Metabólicas Asociadas (CIBERDEM)—Instituto de Salud Carlos III, Madrid, Spain; 3 Institute for Research in Biomedicine (IRB Barcelona), The Barcelona Institute of Science and Technology, Barcelona, Spain; 4 Departament de Bioquímica i Biomedicina Molecular, Facultat de Biologia, Universitat de Barcelona, Barcelona, Spain; 5 Health Sciences Research Institute Germans Trias i Pujol (IGTP)—Institute of Predictive and Personalized Medicine of Cancer (IMPPC), Badalona, Spain; 6 Department of Physiological Sciences II, Faculty of Medicine—University of Barcelona, Hospitalet del Llobregat, Barcelona, Spain; 7 LaCell LLC and Center for Stem Cell Research and Regenerative Medicine, School of Medicine, Tulane University, New Orleans, LA USA

**Keywords:** Obesity, Mitochondria, Translational research

## Abstract

**Background:**

A functional population of adipocyte precursors, termed adipose-derived stromal/stem cells (ASCs), is crucial for proper adipose tissue (AT) expansion, lipid handling, and prevention of lipotoxicity in response to chronic positive energy balance. We previously showed that obese human subjects contain a dysfunctional pool of ASCs. Elucidation of the mechanisms underlying abnormal ASC function might lead to therapeutic interventions for prevention of lipotoxicity by improving the adipogenic capacity of ASCs.

**Methods:**

Using epigenome-wide association studies, we explored the impact of obesity on the methylation signature of human ASCs and their differentiated counterparts. Mitochondrial phenotyping of lean and obese ASCs was performed. *TBX15* loss- and gain-of-function experiments were carried out and western blotting and electron microscopy studies of mitochondria were performed in white AT biopsies from lean and obese individuals.

**Results:**

We found that DNA methylation in adipocyte precursors is significantly modified by the obese environment, and adipogenesis, inflammation, and immunosuppression were the most affected pathways. Also, we identified *TBX15* as one of the most differentially hypomethylated genes in obese ASCs, and genetic experiments revealed that *TBX15* is a regulator of mitochondrial mass in obese adipocytes. Accordingly, morphological analysis of AT from obese subjects showed an alteration of the mitochondrial network, with changes in mitochondrial shape and number.

**Conclusions:**

We identified a DNA methylation signature in adipocyte precursors associated with obesity, which has a significant impact on the metabolic phenotype of mature adipocytes.

## Introduction

Obesity has become a major public health concern as it is a risk factor for several diseases, including type 2 diabetes (T2D), cardiovascular diseases and some forms of cancer. It is now recognized that obesity, which has been traditionally viewed as arising primarily from an imbalance between energy intake and expenditure favoring fat storage, results from interactions between environmental and genetic factors. Indeed, a growing body of evidence supports epigenetics as a key mechanistic mediator of gene−environment interactions underlying obesity and its comorbidities [1].

White adipose tissue (WAT) is conceivably the most plastic organ in the body. In response to a chronic positive energy balance, WAT expands by increasing the volume of preexisting adipocytes (hypertrophy), and by generating new adipocytes (hyperplasia) through the recruitment of adipocyte progenitors termed adipose-derived stromal/stem cells (ASCs), which reside in perivascular WAT stroma. ASCs undergo adipogenesis and differentiate into mature adipocytes to maintain adipocyte turnover under a normal metabolic state [2]. Available information suggests that WAT homeostasis is perturbed in an obesogenic context because of dysregulated adipogenesis [3], supporting the concept that ASCs are important players in WAT remodeling during obesity [4].

We and others have demonstrated that subcutaneous adipose tissue (SAT) from obese subjects contains a dysfunctional pool of human ASCs (hASCs). Accordingly, there is evidence for an association between obesity and loss of hASC multipotency [5], increased hASC proliferation [6], resistance to apoptosis [7], and reduced adipogenic potential [6, 8]. Moreover, a recent study from our group showed that the metabolic phenotype of the donor compromises the immunomodulatory properties of hASCs [9].

Stem cell differentiation is guided by epigenetic processes that confer a specific chromatin conformation on the genome [10]. Among the best characterized of these are post-translational modifications of histone tails and CpG dinucleotide methylation. Adult stem cells of different origin (e.g., WAT and bone marrow) exhibit a range of differentiation potentials to mesodermal, endodermal, and ectodermal tissues, and strong methylation of lineage-specification promoters restricts their ability to differentiate, underlining the existence of specific epigenetic profiles associated with different degrees of differentiation potential [11]. Alterations in DNA methylation during differentiation/adipogenesis of hASCs, either under physiological or pathological conditions, have been poorly explored. Indeed, only a few adipogenic genes have been examined for methylation of CpG islands to date [12], and only one study has reported the DNA methylation profile of hASCs during differentiation, specifically osteogenic and myogenic differentiation [11]. We hypothesized that an obese environment would influence the methylation status of genes in hASCs, which might contribute to the contrasting differentiation and functional capacities of adult adipocytes to promote dysfunctional WAT that is associated with enhanced susceptibility to certain comorbidities such as T2D [13]. In line with this, it has been recently described that epigenetics plays a role in the development of adipose tissue dysfunction in obesity [14]. Here, using epigenome-wide association studies, we show that whereas the DNA methylation signature remains relatively static during the transition of hASCs to fully mature adipocytes, it is significantly modified by an obese environment, supporting the view that hASC dysfunction is a key regulatory event in obesity. We also demonstrate that the methylation status of *TBX15*, a transcription factor of the T-box family involved in adipogenesis, fat distribution and browning [15–17], might play an essential role in the mitochondrial phenotype of WAT in obesity.

## Materials and methods

### Study subjects

For epigenetic-wide association studies (EWAS), hASCs were obtained from SAT of healthy female donors undergoing elective liposuction surgery (cohort I): *n* = 6 lean, BMI 22.4 ± 12 kg/m^2^, age 44.3 ± 9.2 years; *n* = 6 obese, BMI 32.6 ± 2.2 kg/m^2^, age 34.3 ± 7.4 years (LaCell LLC, New Orleans). Donors were classified as lean or obese based on BMI following World Health Organization criteria. All participants gave their informed consent and the study was reviewed and approved by the Western Institutional Review Board (Puyallup, WA, USA; Protocol # 201304490).

For other molecular analyses, hASCs were isolated from SAT of age- and sex-matched donors undergoing nonacute surgical interventions, such as hernia or cholecystectomy, in a scheduled routine surgery (cohort II): *n* = 4 lean, BMI 23.7 ± 1.1 kg/m^2^, age 52.8 ± 11.5 years; *n* = 4 obese, BMI 32.4 ± 3 kg/m^2^, age 52 ± 10.5 years. All participants gave their informed consent and the study was reviewed and approved by the ethics and research committee of University Hospital Joan XXIII, Tarragona, Spain.

### DNA methylation profiling using universal bead array

Genomic DNA was extracted from cells using the NucleoSpin® Tissue Kit (Macherery-Nagal GmbH). DNA methylation profiles were generated on the Infinium Human-Methylation450K BeadChip (Illumina). The BeadChip was developed to assay more than 480,000 CpG sites and selected CpG loci in parallel. DNA methylation data were processed using GenomeStudio software (Illumina) by applying the default settings. Methylation data is in the GEO database with the accession code *GSE111632*.

### Methylation data analysis

All computations and statistical analyses were performed using R 3.0.2 and Bioconductor 2.13. Analysis of the beadchip was performed using BiSeq 1.8.0 package [18], which involved the following stages: creation of an object of the Bssraw class (container of the raw data), CpG cluster identification, methylation ratio smoothing in each CpG cluster identified, test and model group effect for each CpG site inside of CpG clusters, test CpG clusters for differential methylation and identification of differentially methylated sites (DMSs) [19]. Smoothing criteria was performed using 90% quantiles, and CpG clusters considered for DMS identification were filtered using a false discovery rate <10% and with at least one differentially methylated position. The β value, which represents the percentage of methylation at each CpG site, was calculated from the normalized intensity value. The Δβ or log 2-fold change values at all CpGs sites from the different comparisons (undifferentiated minus differentiated state or lean minus obese condition) were analyzed using unpaired Student’s *t* test and results with *p* values < 0.0001 were further investigated.

### Gene ontology analysis

Gene ontology (GO) analysis of the selected differentially methylated regions was performed using Ingenuity Pathway Analysis (IPA, Qiagen) and Panther [20].

### Gene expression analysis

Total RNA was extracted from hASCs using the RNeasy Mini Kit (Qiagen). One microgram of RNA was reverse transcribed with random primers using reverse transcriptase (Applied Biosystems). Quantitative gene expression was evaluated by qPCR on a 7900HT fast real-time PCR system using hydrolysis probes gene expression assays (Supplementary Table S1). The relative expression of each transcript was measured using the 2^−ΔΔCt^ method. Endogenous 18s RNA was used for normalization of gene expression levels and calculation of ΔCt values. Values are expressed as mean ± SEM. Data were analyzed using Excel and GraphPad Prism software 5. Differences were considered statistically significant if the *p* value was <0.05 (*t* test).

### Statistical analyses

The sample size of the epigenetic study was calculated to detect differences for methylation levels taking into account published values of epigenome-wide analysis in the field of stem cells [11, 21]. Data are presented as mean (SEM) or median (25th–75th) quartiles as appropriate. Before statistical analysis, normal distribution and homogeneity of the variances were evaluated using Levene’s test. Student’s paired two-sample *t* test and NPAR test (Wilcoxon) were used for anthropometric and biochemical variables. Mann−Whitney test was used to compare groups of adipose tissue samples and Student’s paired two-sample *t* test was used for the in vitro data. All experimental results were from 3−4 independent experiments performed at least in duplicate. The relationship between quantitative variables was tested using Pearson’s test and correlations with Spearman’s analysis. Statistical analyses were performed using the program SPSS (version 15.0).

## Results

### Impact of obesity on global DNA methylation patterns in hASCs and differentiated adipocytes

**Fig. 1:**
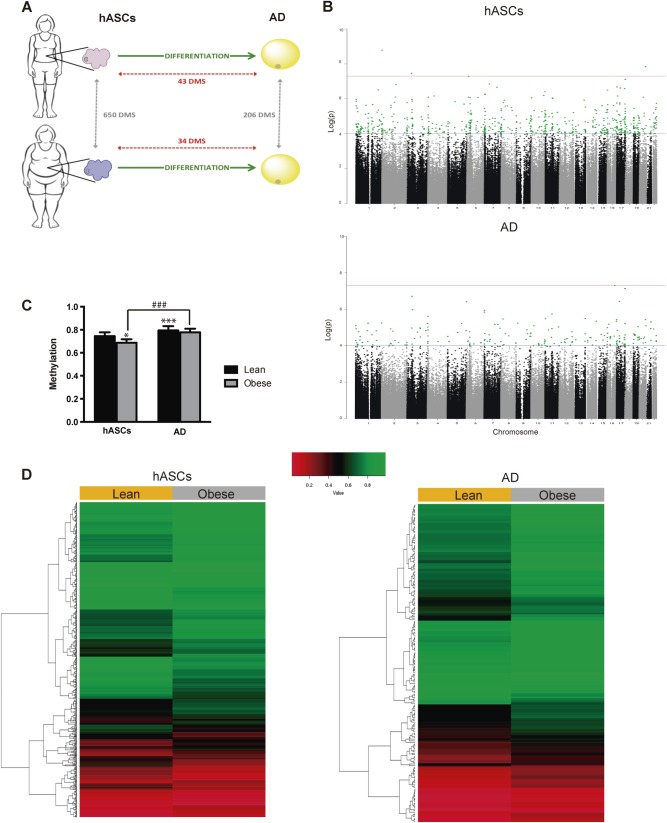
Obesity modifies the hASC methylome. **a** Schematic representation of the approach performed and the main results obtained (*p* < 0.0001). **b** Manhattan plots (−Log_10_(*p*) genome-wide association plot). All DMSs achieving the significance threshold are colored in green (*p* < 0.0001). **c** Global methylation status of ASCs and mature counterparts. Results are shown as mean ± SEM. *t* test; *p < 0.05, ***p < 0.001 vs. lean-derived hASCs; ###*p* < 0.001 vs. obese-derived hASCs. **d** Supervised cluster analysis and heat map showing the differential groups of CpGs according to their methylation profile in all analyzed samples. *n* = 6, patients per group. hASCs human adipose-derived stem cells, AD adipocyte, DMS differentially methylated site (color figure online)

As a first step to investigate the potential contribution of obesity to DNA methylation and adipocyte differentiation, hASCs were isolated from the SAT compartment of lean and obese subjects (*n* = 6; as described in Materials and methods). Isolation and differentiation of hASCs to mature adipocytes was performed as described [6, 9]. We extracted genomic DNA from cells before and after differentiation and performed EWAS on the Illumina platform using the Infinium Human-Methylation450K BeadChip array (Fig. 1a). We established a threshold of log 2-fold change with a *p* value < 0.0001 to discern DMSs between the conditions. At the global level, only 0.01% of the CpG sites studied displayed changes between differentiation states, whereas this rose to 0.2% when lean and obese conditions were considered. We identified 650 DMSs between hASCs from lean and obese subjects and only 206 DMSs between equivalent mature adipocytes (Fig. 1a, b), with a total of 64 DMSs in common. In lean subjects, 43 DMSs were identified between hASCs and differentiated adipocytes, whereas 34 DMSs were identified in obese subjects (Fig. 1a). Overall, these data show that the methylome is comparatively stable between hASCs and mature adipocytes, and also that the majority of the differences detected are due to the obese environment already established in the hASC niche. The apparent discordance between the number of DMSs in hASCs and adipocytes according to phenotype (lean vs. obese) can be explained because of the use of an arbitrary cutoff (see Materials and methods), which could have led to small changes not identified as statistically different DMSs. Nevertheless, *β* value differences between lean and obese donors showed a strong correlation for DMSs both in ASCs (*n* = 650) and in adipocytes (*n* = 206), but not for a subset of randomly selected probes (*n* = 1000) (Supplementary Figure S1). This indicates that DMSs between lean and obese are largely maintained in both ASCs and adipocytes, even when the *p* value does not reach the threshold (0.0001). Also, since global methylation is known to increase during differentiation, this may have conceivably masked small changes occurring during the differentiation of hASCs to mature counterparts. Interestingly, global methylation levels were significantly lower in obese-derived hASCs than in lean-derived counterparts (Fig. 1c). Moreover, 88.37% (38 from 43 DMSs) of the methylation changes that occurred during adipogenesis were hypermethylation events. We next analyzed hierarchically all the CpG methylation differences observed between lean and obese states. Supervised cluster analysis and heat map analysis revealed that the CpG sites analyzed segregated into two clusters, and indicated that only a small proportion of the CpG sites had a low degree of methylation (Fig. 1d).

**Fig. 2:**
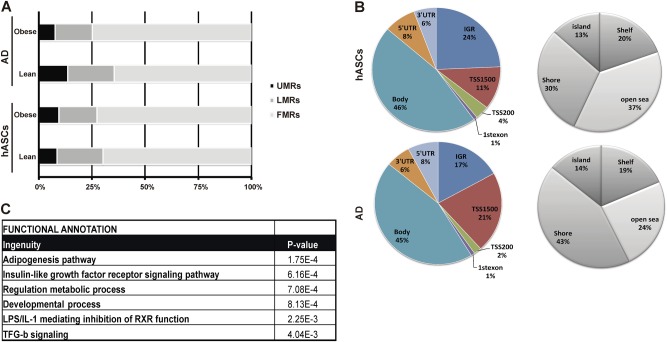
DNA methylation changes during adipocyte differentiation. **a** The percentage of DMSs distributed into three classes: fully methylated regions (FMRs > 50% methylated CpGs); low methylated regions (LMR, 13.9–50%); and unmethylated regions (UMRs < 13.9%). **b** DMS distribution and CpG allocation. **c** Pathway analysis for genes associated with DMSs in hASCs

We next segmented the four methylomes into three classes: fully methylated regions (FMRs; >50% methylated CpGs), low methylated regions (LMRs; <13.9–50%) and unmethylated regions (UMRs; <13.9%), as previously described [22, 23]. The FMR class comprised over 50% of the methylome in all samples (Fig. 2a), and the global percentage of methylation was stable between lean and obese hASCs. By contrast, when we compared adipocytes differentiated from hASCs of lean and obese subjects, we found a global increase in methylation in the latter, with an important gain in FMRs (overall increase of 16%) and a decrease in the number of UMRs. Several studies suggest that the effects of DNA methylation on gene expression are highly dependent on the genomic location of the CpG dinucleotide [22, 24]. To assess whether differential methylation is preferentially allocated at specific genomic regions, we classified all DMSs into seven categories of genome annotations: located in promoters–transcription start site (TSS) (a) −200 bp (TSS200) and (b) −1500 bp (TSS1500); (c) intergenic region (IGR); located in intragenic regions (d) 1st exon, (e) gene body, (f) 3′UTR, and (g) 5′UTR. The majority of changes found in hASCs were those located in transcribed regions (body), which have been shown to positively correlate with transcribed genes [24] (Fig. 2b, left panel). While a similar tendency was observed in mature adipocytes, we detected an approximate twofold increase in the location of DMSs at distal promoters (TSS1500) where DNA methylation is normally negatively correlated with gene expression (Fig. 2b, left panel). The DMS distribution within a CpG island (island>shelf>shore>open sea) is depicted in Fig. 2b, right panels. In both settings, 67% of the significant changes were found in the most distal areas from a CpG island (shore and open sea).

Overall, the above data show that hASCs are obesity-conditioned through the accumulation of epigenetic modifications, at least at the level of DNA methylation, which might underlie the altered plasticity of obese-derived hASCs as previously reported by us [6, 9] and others [8, 25]. In an attempt to discern which molecular pathways might be influencing the hASC niche in an obese environment, we performed a functional analysis by GO enrichment using the Ingenuity database for all genes containing DMSs. Since we were specifically interested in the functionality of adipocyte precursors as key determinants of adipose tissue expansion, we focused on changes in the hASC compartment. As anticipated, GO analysis identified gene functions associated with adipogenesis (Fig. 2c). Other differentially enriched biological pathways detected included inflammation (LPS/IL-1) and immunosuppression/repair (TFG-β). These findings are in agreement with our earlier studies showing that hASCs from obese patients have a heightened inflammatory profile and reduced immunosuppressive properties [6, 9], and are consistent with results from other independent studies [26, 27].

### Influence of epigenetic signature on gene expression in hASCs

**Fig. 3:**
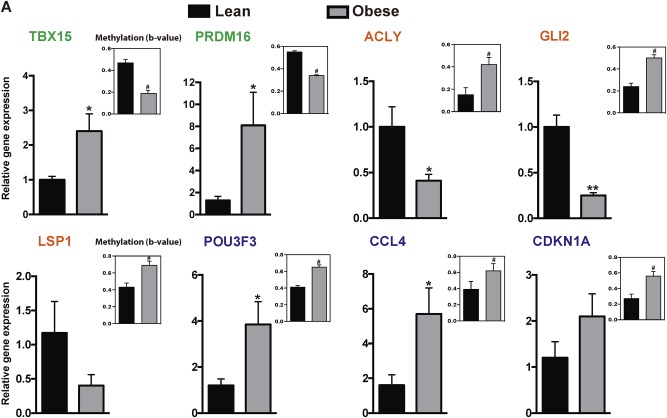
Differentially methylated sites have a differential effect on mRNA expression. **a** Methylation and mRNA levels of *TBX15*, *PRDM16*, *ACLY*, *GLI2*, *LSP1*, *POU3F3*, *CCL4*, and *CDKN1A* genes in hASCs. Significant DMSs (#*p* < 0.0001) identified by EWAS are shown (*n* = 6 patients per group). Verification of mRNA levels of genes associated with the DMSs identified (*n* = 4 patients per group). Results are shown as mean ± SEM. *t* test; *p < 0.05, **p < 0.01 vs. lean hASCs. Gene name in green (decreased methylation and increased *mRNA* expression), in orange (increased methylation and decreased *mRNA* expression), and in blue (methylation and *mRNA* expression incongruity) (color figure online)

To explore in more detail the methylation changes in lean- and obese-derived hASCs, we first used a more restrictive cutoff to exclude all sites with less than 20% methylation change and to give us a more manageable number, which reduced the list from 650 DMSs to 60. The top ten genes with the greatest DNA methylation changes (hyper- and hypomethylated) are shown in Table 1. DNA methylation is most often linked to gene silencing. From the eight genes screened to evaluate the correlation between methylation status and mRNA expression, the expected inverse relationship between gene expression and methylation level was established for five of them: *TBX15*, *PRDM16*, *ACLY*, *GLI2*, and *LSP1* (Fig. 3a). In the remaining three genes (*POU3F3*, *CCL4*, *CDKN1A*), an increase in methylation in obese hASCs was associated with higher mRNA levels (Fig. 3a). As the genomic location of DMSs might contribute to explain these divergences, we next examined this finding that DNA methylation around promoter and intragenic regions negatively correlated with gene expression (Table 1 and Fig. 3a). For example, *TBX15* was demethylated to different degrees in obese hASCs as compared with lean counterparts, which was accompanied by upregulation at the mRNA level. By contrast, DMSs around the IGR did not trigger a direct modification at the mRNA level. This was the pattern observed for *CCL4*, *CDKN1A*, and *POU3F3* in which the occurrence of methylated DMSs located in the IGR (Table 1) did not correlate with downregulation of the mRNA (Fig. 3a). For the transcription factor *GLI2*, we detected an increase in methylation above 20% within an IGR; nevertheless, expression of this gene was significantly decreased in obese hASCs. This could be explained by the finding that *GLI2* possesses other significant DMSs in the gene body whose DNA methylation levels were in agreement with its pattern of mRNA expression (Supplementary Table S2).

**Table 1:** Top 10 hypo- and hyper-methylated sites with more of 20% of change

### Hypomethylation of mesodermal developmental gene *TBX15* in obese hASCs is associated with mitochondrial function in mature adipocytes

**Fig. 4:**
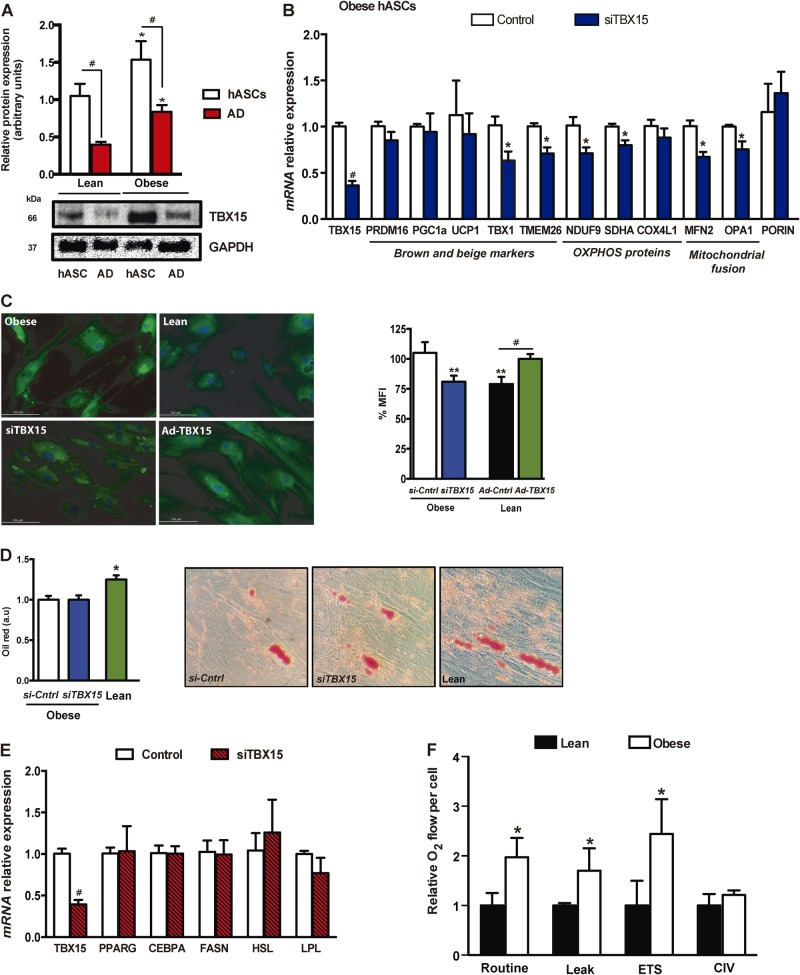
Obesity impacts mitochondrial functionality in hASCs and in differentiated adipocytes. **a** TBX15 protein levels in hASCs from lean and obese subjects and in differentiated adipocytes (AD) (*n* = 3 patients per group). *t* test; #*p* < 0.05 vs. hASCs; *p < 0.05 vs. lean cells. **b** hASCs isolated from obese subjects were transfected with 100 nM of siRNAs against *TBX15* or control, followed by quantitative PCR (qPCR) analysis of the expression of brown and beige markers, mitochondrial redox carriers and mitochondrial fusion genes (*n* = 4 patients per group). *t* test; #*p* < 0.001 and *p < 0.05 vs. control cells. **c** Mature adipocytes derived from obese *TBX15*-silenced hASCs or lean hAScs overexpressing *TBX15* were analyzed using Mitotracker staining by flow cytometry; representative images are shown (*n* = 3) *t* test; **p < 0.01 vs. obese control; #*p* < 0.05 vs. lean control. **d** Quantification and representative intracellular lipid enrichment in adipocytes derived from obese, obese *TBX15*-silenced and lean individuals (magnification, ×20). *t* test; *p < 0.05 vs. control obese cells. **e** Gene expression analysis of adipogenic markers by qPCR in mature obese adipocytes (*n* = 4 patients per group). *t* test; #*p* < 0.001 vs. control obese cells. **f** Oxygen consumption in intact adipocytes was measured by respirometry (*n* = 6 patients per group). Results are shown as mean ± SEM. *t* test, *p < 0.05 vs. lean

We focused on *TBX15* since it was highly regulated at the epigenetic level, containing a total of 13 DMSs (Table 1 and Supplementary Figure S2). *TBX15* belongs to the T-box family of homeodomain transcription factors, which are essential for many developmental processes. The methylation levels of *TBX15* were further validated by bisulfite sequencing, confirming that the amplicon was demethylated in obese hASCs (Supplementary Figure S3). The high mRNA level of *TBX15* in obese hASCs (Fig. 3a) was confirmed at the protein level (Fig. 4a). Intriguingly, whereas the protein levels of TBX15 were higher in obese hASCs than in lean hASCs, its expression significantly decreased throughout the adipogenic differentiation process both in obese- and lean-derived cells, suggesting a developmental role for TBX15 in WAT adipogenesis. Even so, TBX15 protein levels remained higher in mature adipocytes from obese-derived hASCs than from those of lean subjects. A previous study in murine adipocyte precursors indicated that Tbx15 regulates brown but not white adipocyte differentiation [17], whereas another study described Tbx15 as a negative regulator of mitochondrial mass and adipogenesis in murine 3T3-L1 pre-adipocytes [15]. To explore the effects of TBX15 on adipocyte precursors, we first transfected obese-derived hASCs with a pool of three target-specific siRNAs against *TBX15* to silence its expression. TBX15-silenced cells showed a significant decrease in the expression of beige adipocyte markers (including *TBX1* and *TMEM26*), catalytic components of mitochondrial complex I (*NDUFA9*) and II (*SDHA*), and mitochondrial fusion markers (including *MFN2* and *OPA1*) (Fig. 4b), in part, supporting the proposed role of TBX15 in the beiging of SAT [17]. Remarkably, most of these genes were upregulated in obese ASCs as compared with equivalent lean cells (Supplementary Figure S4) which, considering the higher levels of *TBX15* in obese ASCs, supports its instructive role in the mitochondrial phenotype of adipocytes. Additionally, we detected a decrease in the expression of genes encoding proteins for fatty acid transport and oxidation (*ACAA1*, *CPT1A*, *CPT1B*, *SLC25A20*), glucose uptake (*GLUT4*), glycolysis (*LDHA*, *LDHB*) and the TCA cycle (*OGDH*, *PDH*) in *TBX15*-silenced obese ASCs (Supplementary Figure S5). Interestingly, most of these genes were also found increased in obese compared with lean ASCs [9]. Overall, these results suggest a potential role for TBX15 in the mitochondrial and/or metabolic phenotype of mature adipocytes in an obesity context. Accordingly, downregulation of *TBX15* expression in obese precursors before inducing differentiation resulted in a decrease in the mitochondrial mass of mature adipocytes, pointing to a possible reversal of the obese phenotype (Fig. 4c). Further, silencing TBX15 expression did not affect adipocyte differentiation, at least in terms of lipid content (Fig. 4d) and expression of typical adipogenic markers (Fig. 4e). Conversely, when we increased TBX15 expression in lean-derived ASCs with an adenovirus vector, cells showed an upregulation of mitochondrial marker expression (Supplementary Figure S6), and this was accompanied by an increase in mitochondrial mass in lean-derived differentiated adipocytes (Fig. 4c). Although we failed to detect significant changes in mitochondrial mass or respiratory capacity between obese- and lean-derived hASCs (Supplementary Figure S7), mature adipocytes from obese-derived hASCs exhibited a higher mitochondrial respiratory capacity than those from equivalent lean-derived cells (Fig. 4f). Overall, these results indicate that TBX15 expression in human adipocyte precursors establishes the mitochondrial phenotype of mature cells.

### Mitochondrial phenotype of human subcutaneous adipose tissue in obesity

**Fig. 5:**
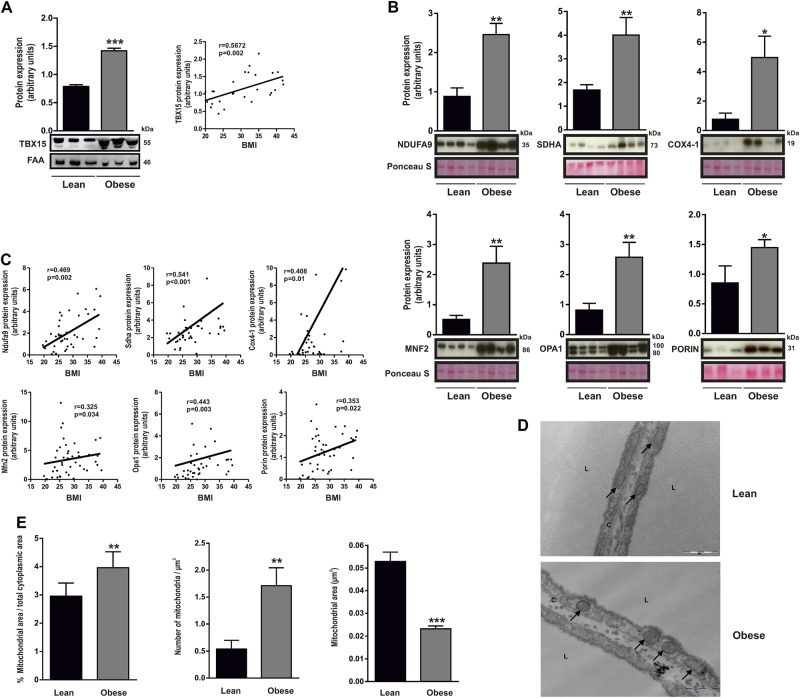
Mitochondrial phenotype of human subcutaneous adipose tissue in obesity. **a** SAT of lean and obese subjects was subjected to immunoblotting against TBX15. Representative immunobloting and quantification is shown (*n* = 14 patients per group). Correlation (Spearman’s analysis) between BMI and TBX15 protein expression is also shown. **b** Mitochondrial fractions isolated from SAT of lean and obese patients were subjected to immunoblotting with antibodies against complex I (NDUFA9), complex II (SDHA), complex IV (COX4-1), MFN2, OPA1, and PORIN. Ponceau S was used as a loading control. A representative blot and densitometry analysis of 11 lean and 32 obese patients performed is shown. **c** Correlation (Spearman’s analysis) between BMI and complex I (NDUFA9), complex II (SDHA), complex IV (COX4-1), MFN2, OPA1, and PORIN. **d** Transmission electron microscopy images of mitochondria in SAT from lean and obese subjects. Representative images are shown. Scale bars, 500 nm. Black arrows indicate mitochondria; C Cytoplasm, L Lipid droplet. **e** Quantification of absolute mitochondrial area vs. total cytoplasmatic area, number of mitochondria and mitochondrial area (*n* = 3 patients for each group). Values are expressed as mean ± SEM. Mann−Whitney test; *p < 0.05; **p < 0.01; ***p < 0.001 vs. lean

To extend this analysis, we examined TBX15 expression in SAT from lean and obese subjects. Several studies have demonstrated obesity-triggered mitochondrial dysfunction in WAT, both in clinical subjects and experimental models [28, 29], but whether this is a cause or a consequence of the disease process is unclear. Moreover, contrasting reports have been published regarding depot-specific and obesity-dependent expression of TBX15 [15, 16, 30], and its potential modulation of mitochondrial metabolism [15, 16, 30]. Expression analysis of TBX15 in SAT from individuals classified according to BMI (clinical and laboratory data summarized in Supplementary Table S3) revealed significantly higher TBX15 protein levels in obese than in lean subjects (Fig. 5a). Consistent with the ability of TBX15 to regulate the mitochondrial phenotype (Fig. 4), higher TBX15 expression in obese SAT correlated with a significant increase in the expression of several mitochondria-related genes such as the oxidative phosphorylation subunits NDUFA9 (complex I), SDHA (complex II) and COX4–1 (complex IV) (Fig. 5b). Interestingly, the expression of the mitochondrial fusion proteins MFN2 and OPA1 was also higher in mitochondria fractions of SAT from obese subjects (Fig. 5b). We also detected a significant increase in the expression of porin, a mitochondrial mass indicator. Accordingly, expression of these proteins was positively associated with BMI (Fig. 5c). Finally, we examined mitochondrial morphology in SAT samples by transmission electron microscopy (Fig. 5d). Results showed that adipocytes from obese subjects had significant alterations in mitochondrial morphology and size as compared with those of lean individuals. Specifically, mitochondrial number and area were significantly higher in obese-derived SAT than in lean-derived SAT, whereas mitochondrial size was significantly smaller (Fig. 5e). Since mitochondrial function is closely linked both to mitochondrial shape and the intracellular distribution of mitochondria, these changes detected in obese WAT might be directly associated with adipocyte energy disturbances linked to obesity.

## Discussion

DNA methylation is one of the best studied epigenetic mechanisms regulating gene transcription and encodes genetic and environmental effects as stable chemical modifications of DNA. A better understanding of the epigenetics of hASCs might help in the development of new strategies designed to combat obesity and/or promote “healthy” WAT expansion. In the present study, we show that the differentiation of hASCs into mature adipocytes is reflected in a discrete number of changes in DNA methylation. By contrast, the methylation signature of obese and lean hASCs is widely different, presenting an unprecedented epigenetic model for the general short circuit in the stemness network of obese hASCs [5]. Accordingly, an obese environment may imprint a specific epigenetic signature on adipocyte precursors so they become dysfunctional adipocytes. Against this background, a previous study from our group revealed that obesity-related adipose tissue hypoxia is necessary but not sufficient to orchestrate significant functional changes in mature adipocytes [6].

Our genome-wide approach allowed us to define new regulatory regions that are differentially methylated in hASCs and that are associated with the control of critical adipogenic differentiation genes. Many of the DMSs identified in the present study were located outside of a proximal gene promoter or CpG island. Conventionally, methylation on promoters blocks transcription whereas methylation in the gene body does not block and might even stimulate transcription [31]. Although the functional roles of gene body DNA methylation are still a matter of debate [32], our results suggest that gene body methylation has a direct impact on gene expression.

Other studies have explored the epigenetic state of hASCs in relation to their differentiation potential, concluding that DNA methylation reflects neither transcriptional status nor potential for gene expression upon differentiation [12, 33]. These studies were, however, performed on a narrow set of adipogenic genes (*LEP, PPARG, FABP4, LPL*), and despite the finding that methylation levels were similar among clones, mRNA levels and hASC differentiation potential to mature adipocytes were heterogeneous [12]. Moreover no consideration was given to other types of stimuli that could epigenetically prime adipose progenitors cells, such as donor metabolic status or obesity, which may explain some of the differences observed between clones. In line with our results, Moskaug and colleagues [34] showed that exposure of hASCs to high glucose upregulated inflammatory genes and altered histone H3 methylation in a manner suggestive of transcriptional de-repression. Clearly, further studies are needed to fully characterize the obesity-linked factors underlying the epigenetic signature of hASCs, as well as the potential reversibility of the process.

Adipose tissue from obese individuals and patients with T2D contains a dysfunctional pool of hASCs [8, 9]. To the best of our knowledge, the present study is the first to establish that the pathological adaptation of hASCs in an obese context is regulated, at least in part, at the epigenetic level. In support of this notion, Ollikainen and colleagues [27] performed an extensive examination of genome-wide DNA methylation in SAT from monozygotic twins with discordant BMI, finding an upregulation of inflammation and extracellular matrix remodeling genes accompanied by a downregulation of adipogenic genes. Although little is known about the molecular mechanisms that go awry in obesity, it is clear that they may be epigenetically regulated prior to disease development. Alterations in SAT are commonly associated with the development of unhealthy obesity, and our study provides evidence for hASC epigenetic dysfunction as a potential key regulatory event in obesity that results in impaired maturation of adipocytes.

Of the 650 CpGs differentially methylated between lean and obese hASCs, 13 were related to the transcription factor *TBX15*. This gene is known to play a major role in mesodermal development in all vertebrates [15]; however, its role in WAT is controversial. Accordingly, it has been described that obesity can both increase [30] and decrease [35] *TBX15* mRNA expression. Additionally, opposing functions have been attributed to *TBX15* with respect to adipogenesis and mitochondrial activity [15, 17].

We show that *TBX15* is hypomethylated at multiple CpG sites in obesity and its expression is upregulated in hASCs and SAT from obese subjects. Moreover, *TBX15* is highly expressed in hASCs and its expression is reduced during differentiation, suggesting an important developmental role. Indeed, Tbx15 depletion studies in murine adipose tissue-derived cells have demonstrated an essential role for Tbx15 in the development of adipogenic and thermogenic programs in adipocytes [17]. By contrast, studies using the murine preadipocyte cell line 3T3-L1 showed that Tbx15 overexpression inhibited adipogenesis and mitochondrial respiration [15]. We show here that TBX15 expression in hASCs directly influences the phenotype of mature adipocytes. Obese-derived hASCs silenced for TBX15 expression have decreased mRNA levels of mitochondrial and metabolic markers and, when induced to differentiate, have a diminished mitochondrial mass. Obese-derived adipocytes showed higher respiratory capacity than lean counterparts and SAT of obese individual displayed an increased mitochondrial mass with a concomitant increase in the expression of several respiratory chain proteins. Although some studies have reported a decrease in the respiratory capacity of SAT in obesity [36, 37], other studies are in agreement with our present results [38, 39]. While it is believed that reduced mitochondrial function is a hallmark of human obesity [28], several considerations need to be taken into account: most of the studies have been performed on isolated mitochondria where the mitochondrial communication network is completely lost, whereas our study used intact cells. Furthermore, although mitochondrial DNA has been extensively used as an index of mitochondrial content, relevant physiological and pathophysiological interpretation of the data could be misconstrued depending on the factor used for normalization, as stated by Dela and colleagues [40]. Additionally, ours is the only study that has used electron microscopy to analyze the mitochondrial network in SAT.

It is important to mention that we examined the effect of mild obesity (mean BMI of 30 kg/m^2^) on mitochondrial function, in which a compensatory response of the fat cell could be occurring. Thus, mild obesity might impair mitochondrial function, but the adipocyte could counteract this by increasing the number of mitochondria. It is known that mitochondria can adapt upon endocrine or metabolic challenge (termed mitochondrial plasticity), to meet their bioenergetic demands. Elongation of mitochondria is a result of increased fusion or decreased fission activity, typical for states of increased energy efficiency (e.g., starvation or senescence). Along these lines and in agreement with our results, shortening of mitochondria is the result of decreased fusion activity or increased fission activity, which is typical for states of reduced bioenergetic efficiency such as an oversupply condition like obesity [41]. Indeed, as obesity induces a switch from glucose to lipid oxidation, a less efficient process in terms of ATP production, it is not unreasonable to consider that in the initial stages of obesity the fat cell attempts to compensate for this by increasing the number of mitochondria.

In conclusion, we show that the methylation status of adipocyte precursors is significantly modified by an obese environment and might determine mitochondrial function of adipose tissue, supporting hASC dysfunction as a key regulatory event in obesity. Overall, our data strongly suggest that although DNA methylation patterns are essentially preserved during adipose tissue lineage commitment, obesity preconditions hASCs with a dynamic loss of DNA methylation in selected regions that may ultimately cause WAT dysfunction and the development of metabolic syndromes in obesity.

## Electronic supplementary material

Supplemental methods

Supplemental tables and figures
