# Supplementary material for: Adipose tissue mitochondrial dysfunction in human obesity is linked to a specific DNA methylation signature in adipose-derived stem cells
Source: Int J Obes (Lond). 2018 Sep 27;43(6):1256–68. doi: 10.1038/s41366-018-0219-6 (PMC6760577; doi:10.1038/s41366-018-0219-6)
Supplement: Supplementary file 1 — Supplemental methods [file 41366_2018_219_MOESM1_ESM.docx]

#### SUPLEMMENTAL METHODS

#### hASC isolation

#### hASCs were isolated from SAT using a standard protocol (1-4). Briefly, SAT was washed extensively with phosphate buffered saline (PBS) and treated with 0.1% collagenase in PBS+1% bovine serum albumin (BSA) for 1 hour at 37ºC with agitation. Digested samples were centrifuged to separate adipocytes from stromal cells. The cell pellet containing the stromal fraction was resuspended in stromal culture medium. The medium was replaced 24 hours after seeding to remove non-adherent cells. When cultures reached approximately 80% confluency at passage 0, the cells were harvested by trypsin, centrifuged for 5 min at 1,200 rpm, and the cell pellet was resuspended at a concentration of 10^6^ hASCs/ml in cryomedium (10% DMSO, 10% DMEM/F-12, 80% bovine calf serum). The cells were dispensed in 1-ml aliquots in cryovials, maintained overnight in a -80°C freezer and then stored in liquid nitrogen until they were required for the individual experiments.

**Cell culture and adipogenic differentiation**

Individual vials of cells were removed from liquid nitrogen, placed in a 37°C water bath and agitated rapidly but gently until the ice crystals were thawed. Cells were transferred to a 15-ml conical tube containing 5 ml of stromal medium consisting of DMEM/F12, 10% FBS, and 1% antibiotics (penicillin and streptomycin). The cell suspension was centrifuged at 1,200 rpm at room temperature, the supernatant aspirated to remove residual DMSO, and the cell pellet resuspended in stromal medium in preparation for plating. Cell suspensions (passage 1) were seeded into T-75 cm^2^ flasks. Cultures were replenished with fresh stromal medium every 2–3 days as previously described (4) and cells were used at passage 3. Flow cytometry analysis of cell marker expression revealed that hASCs showed minimal functional and quantitative criteria as established by the International Society of Cell Therapy (ISCT) and the International Federation for Adipose Therapeutics and Science (IFATS) (Supplemental Table S4). hASCs were differentiated to adipocytes by culturing confluent cells in adipogenic induction medium containing DMEM/F12 supplemented with 10% fetal bovine serum, 1% penicillin/streptomycin, 1 mM dexamethasone, 500 mM IBMX, 33 mM biotin, 5 mM rosiglitazone, 100 nM insulin and 17 mM pantothenate, for 3 days. Cultures were replenished every 3 days with maintenance medium (supplemented medium without IBMX and rosiglitazone) for a period of two weeks (5).

**Bisulfite conversion and direct sequencing**

Bisulfite conversion was performed using 200 ng of DNA with EZ DNA Methylation-Gold^TM^ Kit (ZymoResearch). Converted DNA was eluted in 30 μl. Bisulphite sequencing was performed following Clark´s procedure (6, 7). For each region of interest, PCR amplification was first performed on 1 μl of bisulphite-treated DNA with conventional PCR (Supplemental Table S5). The PCR product was directly used as template for a nested PCR according to each specific primer set. The samples were purified with JETQuick PCR Spin Kit (Genomed) and sequenced with specific primers by GATC Biotech service. Briefly, to assess the DNA methylation state of each cytosine we analyzed the raw sequencing electropherograms visualized with Geospiza FinchTV software. The height of each peak was measured to assess the proportion of each population. For simplicity, the methylation level was ranked in five different intervals 0–0.2, 0.21–0.4, 0.41–0.6, 0.61–0.8 and 0.81–1, which reflected the DNA methylation state of the particular cytosine (8).

**Respiration measurement in intact cells**

Cells were introduced into the 2 ml chamber of an Oroboros-2k™ respirometer (Oroboros® Instruments GmbH) to perform high-resolution respirometry studies. *Routine* respiration was assessed under conventional culture conditions at 37^o^C. To study *Leak* respiration, oligomycin (inhibitor of ATP synthase) was added to the chamber. To study *electron transfer system* (ETS) capacity, carbonylcyanide-p-trifluoromethoxyphenylhydrazone (FCCP) was titrated until maximal respiration was reached. Subsequently, complex I was inhibited by adding rotenone to the chamber, and finally antimycin A was added to inhibit oxidative phosphorylation at the level of complex III. The oxygen consumption that remains is considered *residual oxygen consumption* (ROX). Respiration was stimulated by addition of N,N,N,N´-tetramethyl-1.4-benzenediamine dihydrochloride (TMPD) and ascorbate as substrates for cytochrome c oxidase (complex IV). ROX values were subtracted from the oxygen measurements to calculate oxygen flux in the different respiratory states. Oxygen consumption values were normalized to the total number of cells.

**TBX15 silencing and overexpression**

hASCs were transfected with *TBX15* siRNA (Santa Cruz sc-38477) or a control (Santa Cruz sc-37007) using Lipofectamine 3000 (Life Technologies) diluted in Opti-MEM I Reduced Serum Medium (Gibco), added to the cultured cells for 4 hours. Culture medium was replaced after this period and cells were harvested 2 days after transfection or induced to differentiate the following day. Silencing experiments were also performed in mature adipocytes at day 7 of differentiation.

Adenovirus control or expressing human TBX15 (Vector Biolabs, Malvern, PA
USA) was added to 2 × 10^5^ cells in a 35-mm plate at a multiciplity of infection (moi) of 50, followed by incubation for 2 h at 37ºC in Opti-MEM^®^ Medium (Gibco). After incubation, adenovirus-containing medium was replaced with standard culture medium. Cells were harvested 2 days after infection or induced to differentiate the following day. Overexpression experiments were also performed in mature adipocytes at day 7 of differentiation.

**Isolation of a mitochondrial fraction**

Cells were disrupted mechanically using a Potter homogenizer and mitochondria were isolated by differential centrifugation. Briefly, the cell homogenate was transferred to a 50 ml tube and rinsed with 5–10 ml of STE buffer (250 mM saccharose, 5 mM Tris, 2 mM EGTA, 4% fatty acid-free BSA). The homogenate was centrifuged at 800×g at 4ºC for 10 min to pellet cell debris. The mitochondria-containing supernatant was collected and ultracentrifuged at 10,000×g at 4ºC for 10 min. The pellet from this step was washed once in KHE buffer (120 mM KCl, 5 mM KH2PO4, 3 mM HEPES, 1 mM EGTA) and centrifuged as before. Finally, the mitochondria-containing pellet was resuspended in 100–500 μl of KHE buffer, transferred to a 1.5-ml vial, and stored on ice for subsequent analysis.

**Immunoblot analysis**

Cells and human samples were lysed and homogenized in RIPA buffer containing a protease inhibitor cocktail (Sigma-Aldrich), and protein concentration was determined with the BCA protein assay kit (Pierce Biotechnology). Equal amounts of total protein were separated on SDS-PAGE gels, transferred to Immobilon membranes and blocked. Immunoreactive bands were visualized with SuperSignal West Femto chemiluminescent substrate (Pierce) and images were captured using the VersaDoc imaging system and Quantity One software (Bio-Rad). The following antibodies were used: anti-TBX15 (Santa Cruz; sc-134059), anti-NDUFA9 - complex I (Abcam; ab128744), anti-SDHA - complex II (Abcam; ab137756), anti-COX4-1 - Complex IV (Sigma-Aldrich; SAB2108015), anti-mitofusin-2 (Abcam: ab50838), anti-OPA1 (BD Biosciences; 612606), anti-porin (Calbiochem; PC548); and anti-GAPDH (Thermo Scientific; MA5-15738).

**Mitotracker staining**

For immunofluorescence studies, cells were incubated for 30 min with MitoTracker Red FM (400 nmol/l) and maintained in Krebs solution. Image stacks were captured with a Zeiss AX10 AxioCam MRm microscope. Mitochondrial mass was measured by flow cytometry after loading hASCs with MitoTracker Green FM (400 nmol/l) for 30 minutes. Subsequently, cells were trypsinized and fluorescence was assessed (excitation/emission 488/530) with a FACSARIA III system (Becton Dickinson).

#### Electron microscopy

#### Six samples of SAT from surgery of abdominal hernia from 3 lean subjects (BMI >20 and <25 kg/m^2^) and 3 obese subjects (BMI >30 and <40 kg/m^2^) were cut into pieces of about 1 mm^3^ and transferred to glass vials containing 2% parafomaldehyde and 2.5% glutaraldehyde in phosphate buffer. After 24 h at 4ºC, the samples were washed with the same buffer and post-fixed with 1% osmium tetroxide in the same buffer containing 0.8% potassium ferricyanide at 4ºC. Samples were then dehydrated in acetone, infiltrated with Epon resin for 2 days, embedded in the same resin orientated for longitudinal sectioning and polymerized at 60 ºC for 48 hours. Semithin sections were prepared to corroborate that the sample was good under light microscopy. Ultrathin sections were obtained using a Leica Ultracut UC6 ultramicrotome (Leica Microsystems) and mounted on Formvar-coated copper grids. They were stained with 2% uranyl acetate in water and lead citrate. Sections were observed using a JEM-1010 transmission electron microscope (Jeol, Japan) equipped with a SIS Megaview III CCD camera and AnalySIS software.

#### Morphological analysis

#### Within each adipose tissue sample (n=3 lean; n=3 obese), 50 consecutive 15–20 µm^2^ fields of 40,000× taken from 10 random adipocytes were analyzed. Several mitochondrial parameters were measured on digital electron microscopy images using ImageJ (<http://imagej.nih.gov/ij/>). Pixel measurements were taken to calculate mitochondrial area and cytoplasmatic area. The division between the area occupied by mitochondria (µm^2^) within the total calculated area of ​​cytoplasm (µm^2^) for each adipose tissue type allowed us to calculate the value of the ratio of mitochondria to cytoplasm of adipocytes in each group. We calculated the mean of the area of ​​all the mitochondria to determine the average value (µm^2^) of its surface. Finally, we divided the total number of mitochondria found by the total calculated area of ​​cytoplasm of AT.

**References**

1. Yu G, Wu X, Dietrich MA, Polk P, Scott LK, Ptitsyn AA, et al. Yield and characterization of subcutaneous human adipose-derived stem cells by flow cytometric and adipogenic mRNA analyzes. Cytotherapy. 2010;12(4):538-46.

2. Gimble JM, Guilak F. Differentiation potential of adipose derived adult stem (ADAS) cells. Current topics in developmental biology. 2003;58:137-60.

3. Shah FS, Li J, Zanata F, Curley JL, Martin EC, Wu X, et al. The Relative Functionality of Freshly Isolated and Cryopreserved Human Adipose-Derived Stromal/Stem Cells. Cells, tissues, organs. 2016.

4. Serena C, Keiran N, Ceperuelo-Mallafre V, Ejarque M, Fradera R, Roche K, et al. Obesity and Type 2 Diabetes Alters the Immune Properties of Human Adipose Derived Stem Cells. Stem cells. 2016.

5. Pachon-Pena G, Serena C, Ejarque M, Petriz J, Duran X, Oliva-Olivera W, et al. Obesity Determines the Immunophenotypic Profile and Functional Characteristics of Human Mesenchymal Stem Cells From Adipose Tissue. Stem cells translational medicine. 2016.

6. Clark SJ, Statham A, Stirzaker C, Molloy PL, Frommer M. DNA methylation: bisulphite modification and analysis. Nature protocols. 2006;1(5):2353-64.

7. Patterson K, Molloy L, Qu W, Clark S. DNA methylation: bisulphite modification and analysis. Journal of visualized experiments : JoVE. 2011(56).

8. Forn M, Diez-Villanueva A, Merlos-Suarez A, Munoz M, Lois S, Carrio E, et al. Overlapping DNA methylation dynamics in mouse intestinal cell differentiation and early stages of malignant progression. PloS one. 2015;10(5):e0123263.
