# Supplementary material for: Adipose tissue mitochondrial dysfunction in human obesity is linked to a specific DNA methylation signature in adipose-derived stem cells
Source: Int J Obes (Lond). 2018 Sep 27;43(6):1256–68. doi: 10.1038/s41366-018-0219-6 (PMC6760577; doi:10.1038/s41366-018-0219-6)
Supplement: Supplementary file 2 — Supplemental tables and figures [file 41366_2018_219_MOESM2_ESM.pdf]

Detector	Gene name
**TBX15-**Hs01070089_m1	T-box 15
**PRDM16-**Hs00223161_m1	PR domain 16
**ACLY-**Hs00982738_m1	ATP citrate lyase
**GLI2-**Hs01119974_m1	GLI family zinc finger 2
**LSP1-**Hs00158886_m1	Lymphocyte-specific protein 1
**POU3F3-**Hs00275987_s1	POU class 3 homeobox 3
**CCL4-**Hs99999148_m1	C-C motif chemokine ligand 4
**CDKN1A**-Hs00355782_m1	Cyclin dependent kinase inhibitor 1A
**PGC1a**-Hs00173304_m1	PPARG coactivator 1 alpha
**UCP1**-Hs01084772_m1	Uncoupling protein 1
**GLUT4**-Hs00168966_m1	Glucose transporter type 4
**HK2**-Hs00606086_m1	Hexokinase 2
**PFKM**-Hs00175997_m1	Phosphofructokinase M
**PDK1-** Hs01561847_m1	Pyruvate dehydrogenase kinase, isozyme 1
**PDK4**-Hs01037712_m1	Pyruvate dehydrogenase kinase, isozyme 4
**SDHb**-Hs01042482_m1	Succinate dehydrogenase b
**PDH-** Hs01081518_s1	pyruvate dehyrogenase phosphatase catalytic subunit 1
**OGDH**-Hs01081865_m1	Alpha-ketoglutarate dehydrogenase
**LDHa-** Hs01378790_s1	Lactate dehydrogenase a
**LDHb**-Hs00929956_m1	Lactate dehydrogenase b
**CPT1a-** Hs00912671_m1	Carnitine palmitoyltransferase 1A
**CPT1b**-Hs00189258_m1	Carnitine palmitoyltransferase 1B
**SLC25A20**-Hs00386383_m1	Solute carrier family 25 member 2
**ACAA1**-Hs01576064_g1	Acetyl-CoA acyltransferase 1
**TBX1-** Hs00962558_g1	T-box 1
**TMEM26-** Hs00415619_m1	Transmembrane protein 26
**NDUFA9-** Hs00245308_m1	NADH:ubiquinone oxidoreductase subunit A9
**COX4L1-** Hs00971639_m1	Cytochrome c oxidase subunit 4I1
**OPA1-** Hs01047013_m1	OPA1, mitochondrial dynamin like GTPase
**PORIN-** Hs01631624_gH	Voltage dependent anion channel 1

Supplementary Table S1. Human gene expression analysis. Results were calculated using the comparative Ct method and expressed relative to the expression of the housekeeping genes 18S (Hs03928985_g1).

logFC	p-value	probeID	CHR	gene	Localization
0,2592854	1,57245E-06	cg14624731	2	GLI2	IGR - open sea
0,1097032	0,000382328	cg05403744	2	GLI2	Body - island
0,06061764	0,000641159	cg08561286	2	GLI2	Body - open sea
0,07085017	0,008165607	cg15699099	2	GLI2	IGR - open sea
-0,0189977	0,008590543	cg07855121	2	GLI2	Body - open sea
-0,0273136	0,010291827	cg07133097	2	GLI2	IGR - shore
-0,0337513	0,011179139	cg03253309	2	GLI2	Body - open sea
0,02735958	0,012177626	cg18414033	2	GLI2	IGR - open sea
0,07679371	0,013920025	cg10559151	2	GLI2	Body - open sea
0,0738779	0,016978622	cg21133992	2	GLI2	Body - open sea

Supplementary Table S2. Methylation b-values of the different *GLI2* DMSs from the Infinium 450K CpG array.

	LEAN (n=11)	OBESE (n=32)	p
Age (yr)	53.09 (21.63)	54.06 (12.18)	0.890
Gender (M/F)	8/3	16/16	0.190
BMI (kg/m2)	22.73 (1.77)	30.43 (4.65)	<0.001
Waist (cm)	80 (9.95)	103.71 (7.93)	<0.001
Hip (cm)	93.43 (6.58)	103.44 (12.71)	0.064
SBP (mmHg)	122.60 (13.56)	131.40 (17.44)	0.344
DBP (mmHg)	70.40 (7.63)	72.40 (7.85)	0.648
Total cholesterol (mmol/l)	5.45(1.36)	4.96 (1.04)	0.302
HDL-cholesterol (mmol/l)	1.64 (0.4)	1.36 (0.44)	0.143
Triglycerides (mmol/l)	0.85 (0.74-1.36)	1.06 (0.76-1.47)	0.523
HOMA-IR	1.09 (0.47-1.51)	1.16 (0.74-3.09)	0.355
Glucose (mM)	4.61 (4.44-5.56)	5.44 (4.85-5.69)	0.194
Insulin (µIU/ml)	4.70 (2.36-7.74)	4.49 (2.83-12.58)	0.643

Supplementary Table S3. Anthropometric and biochemical variables. Data are presented as mean (SEM) or median (25th-75th) quartiles as appropriate. T-test pairs and NPAR tests (Wilcoxon)

Antibody	Lean	Obese
**CD34**	0.13±0.1	0.1±0.1
**CD45**	0.5±0.3	0.4±0.3
**CD73**	92.3±5.4	92.9±5.5
**CD90**	93.3±3	95.2±4.3

Supplementary Table S4. Immunophenotypic characterization of the isolate hASCs. The values reflect the mean ± SD percent surface positive staining of hASCs for several surface antigens including hematopoietic and stromal markers (n= 6).

	LSP1	ACLY	PRDM16
**PCR external**			
**Forward**	GGTGTTTATGTTGGGTTGTTATATG	ATTTTTTATGGATTGTTGTTTGGTT	AGTTTTTAAATTGGAAATTTGTATT
**Reverse**	TCAACCCAAAATTCAATTTCATAAT	ACCCTTTCACCCCTTACTCTAATAC	CTACATCTCCCAAAAAACTAAAACC
**PCR Internal**			
**Forward**	TTTTGGTAGTTGTTTATGTATTTTTTTT	GTTTTGAGGTTGAGTTATTGTGTTTT	Forward external
**Reverse**	AAATCAAACAAAATCCCTAAAACTC	CTTACCCACTCTTAACCTCCTCTAAA	AAACACCTAACCCCATAATCAC
	**GLI2**	**TBX15 A**	**TBX15 B**
**PCR external**			
**Forward**	GTTTAGTTGTTGGGAGTGTTTAGAG	AAGATTAGATTTTTTTGGGTTTTTT	GAGATAGGAAAAGTTTTTGTAGAAAG
**Reverse**	CCCATAATTCCTATATAATTTATCAC	ATCTTCCAATCCTAATACCCTAATC	AAAATTCCTTATCCCTAAACTCAAC
**PCR Internal**			
**Forward**	GGAAGTTTTTAAAAATGATGTGGTT	AAAATAATAATAAGAAATTTTAATAGGTTG	TTTTTAATAGGGTAATAGGGTGAATA
**Reverse**	CCCCTTCCCATAACTCCTATATAAT	ATTCTATCCTTTTACAAAAAAACC	Reverse external

Supplementary Table S5. Bisulphite sequencing primers used for validation of the methylation status.

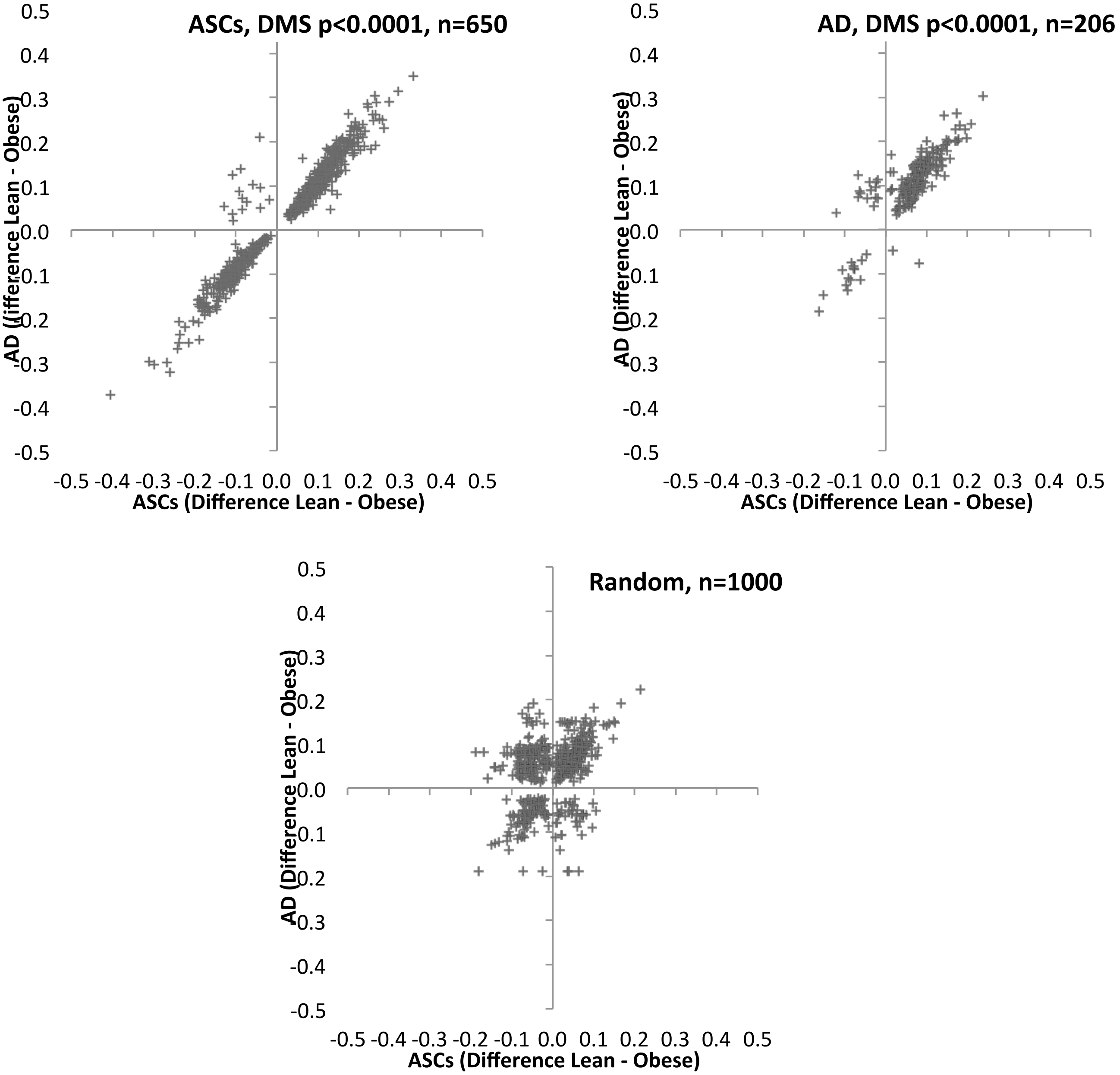

Supplementary Figure S1. Distribution of beta values differences between Lean and Obese by tissue type in Differentially Methylated Sites (DMS) with a p-value<0.0001 in ASCs, AD, ASCs and AD, and random selection of sites (not considering the p-value).

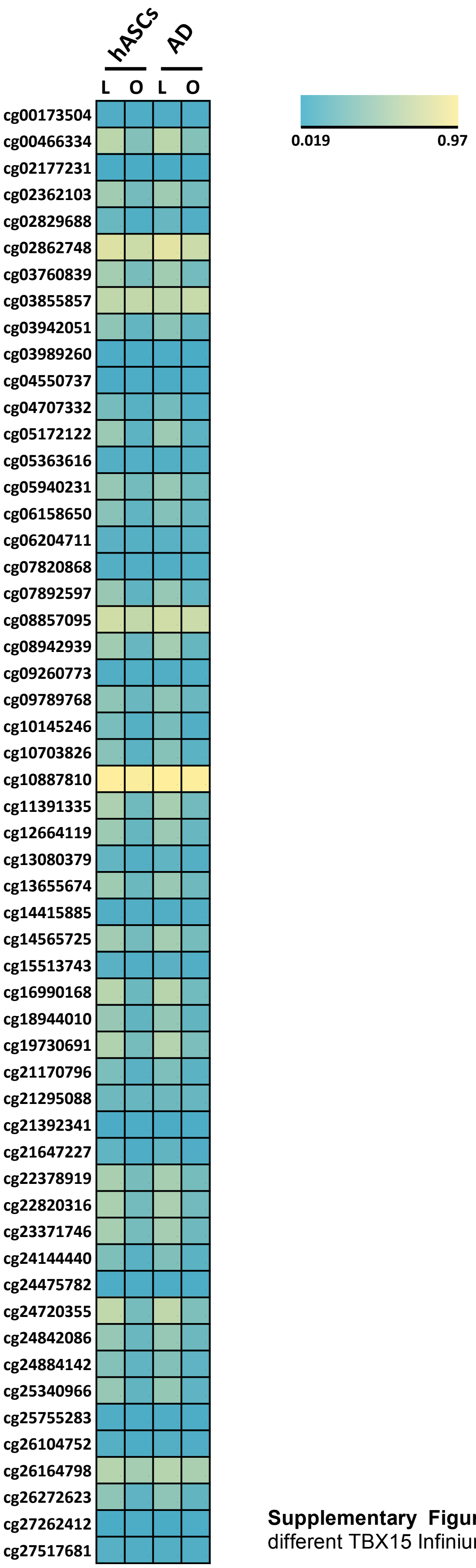

Supplementary Figure S2. Heatmap showing the methylation b-values of the different TBX15 Infinium 450K CpG targets.

Supplementary figure S3

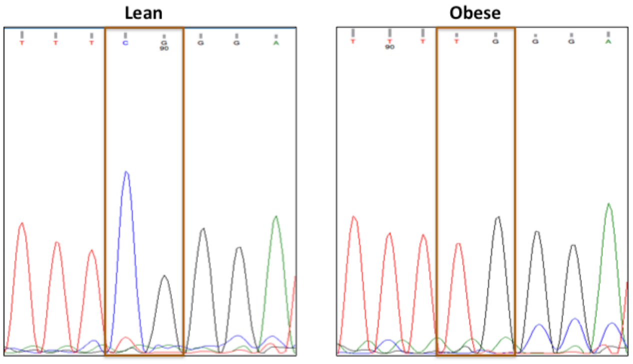

Supplementary figure S3. TBX15 DNA sequencing results following bisulfite treatment. The recovered hASCs DNA was processed using the EZ DNA Methylation Kit, amplified by PCR and sequenced directly. The methylated cytosine (lean) remains intact while the unmethylated cytosine (obese) are completely converted into uracil following bisulfite treatment (detected as thymine following PCR).

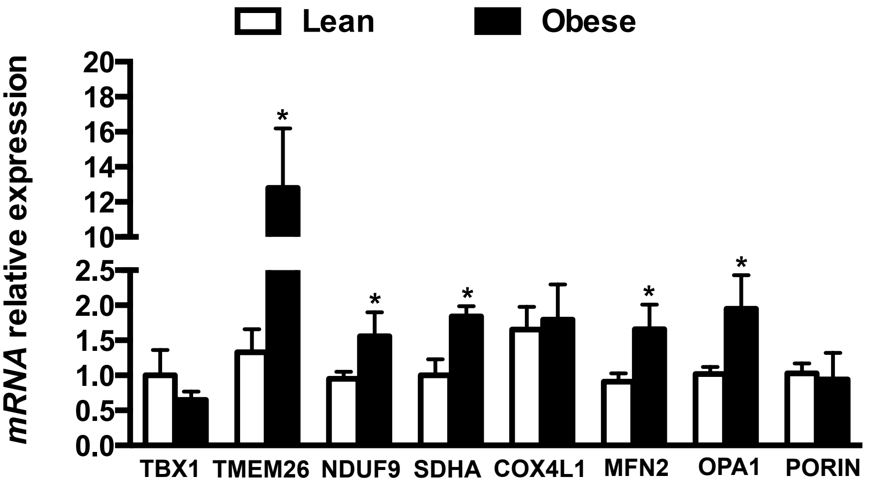

Supplementary figure S4. hASCs isolated from lean and obese subjects were subjected to quantitative PCR (qPCR) analysis of the expression of brown and beige markers, mitochondrial redox carriers and mitochondrial fusion genes (n=4, patients per group). t-test; and *, $p<0.05$ *vs* lean cells

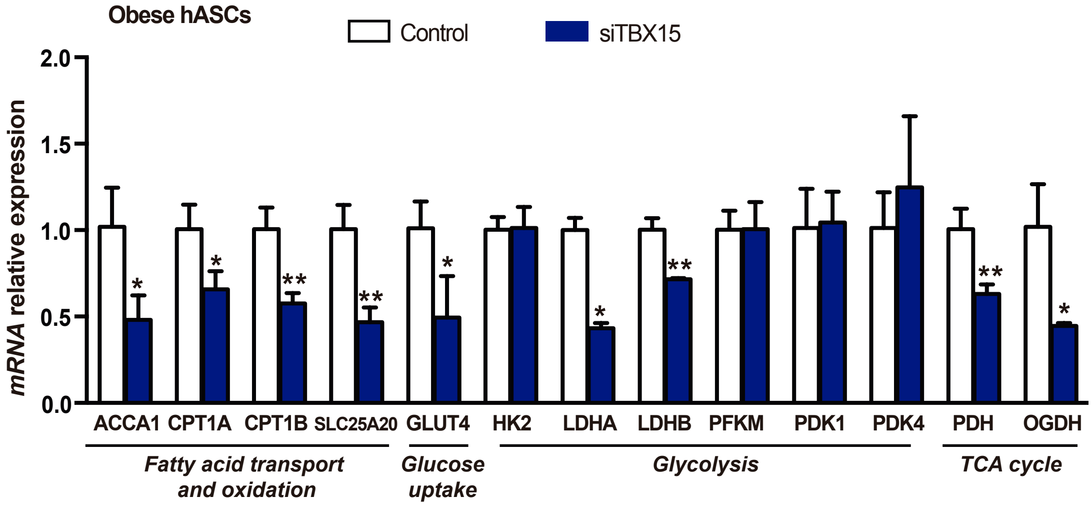

Supplementary figure S5. hASCs isolated from obese subjects were transfected with 100 nM of siRNAs against TBX15 or control followed by quantitative PCR (qPCR) analysis of the expression fatty acid transport and oxidation, glycolysis and TCA cycle genes (n=4, patients per group). t-test; **, $p<0.001$ and *, $p<0.05$ *vs* control cells.

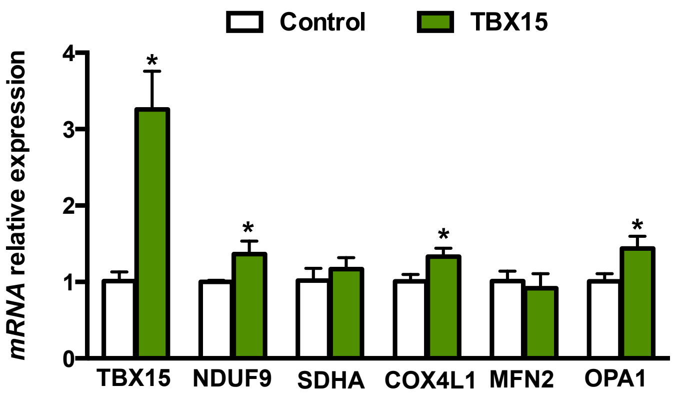

Supplementary figure S6. hASCs isolated from lean subjects were infected with recombinant adenovirus GFP-TBX15 and 48h after infection followed by quantitative PCR (qPCR) analysis of the expression of mitochondrial markers (n=4). t-test; *, $p<0.05$ *vs* control cells.

Supplementary figure S7

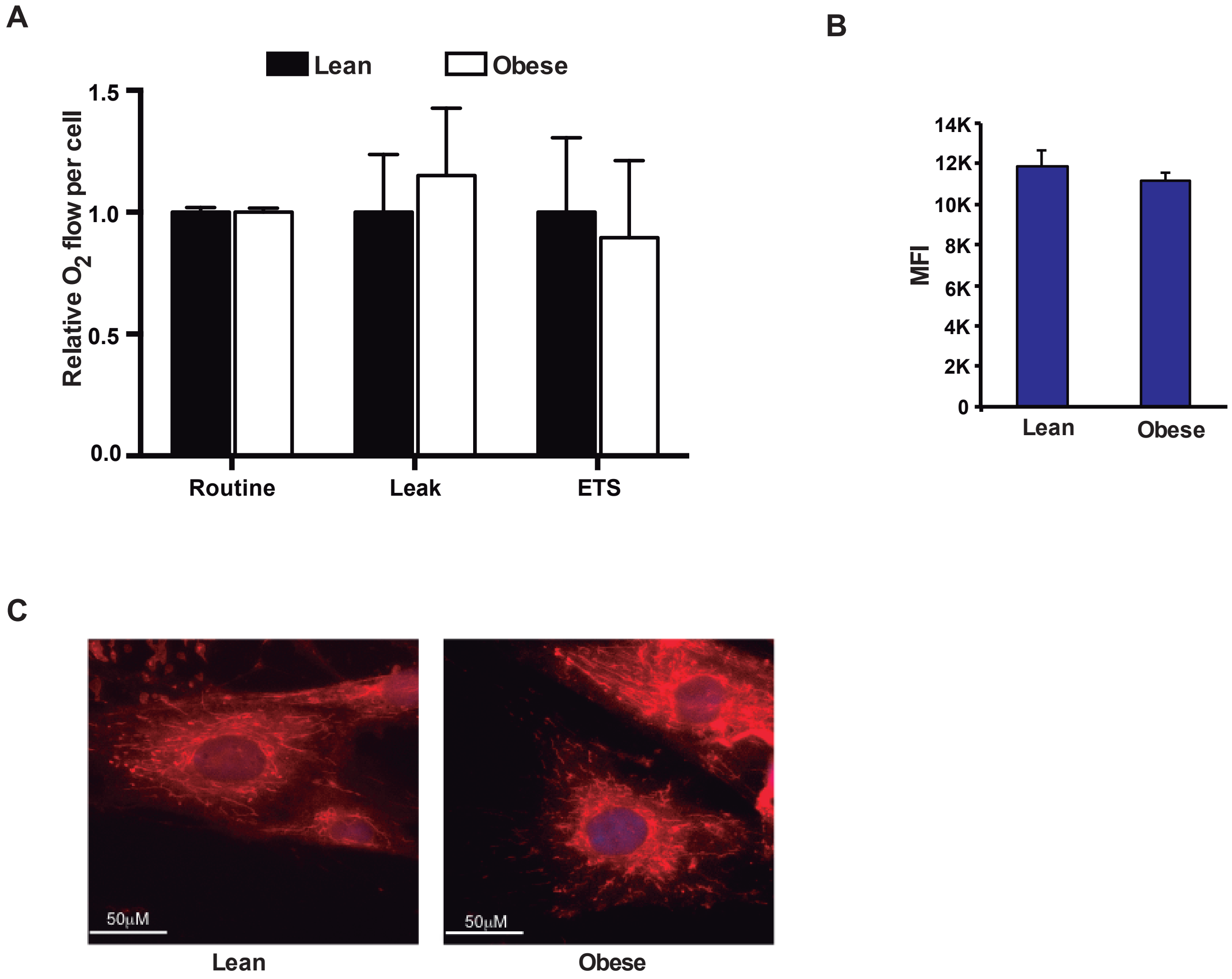

Supplementary Figure S7. Mitochondrial phenotype of hASCs. **A)** Oroboros (Ok2) was used to measure oxygen consumption in intact hASCs. **B)** Mitotracker ® Green FM staining and quantification by flow cytometry in hASCs derived from lean and obese individuals. **C)** Metabolic activity of hASCs derived from lean and obese individuals measured by Mitotracker ® Red CMXRos staining (40x). Values are expressed as mean ± SEM (n=3).
